# Application strategy of finite element analysis in artificial knee arthroplasty

**DOI:** 10.3389/fbioe.2023.1127289

**Published:** 2023-05-17

**Authors:** Zi-Heng Zhang, Yan-Song Qi, Bao-Gang Wei, Hu-Ri-Cha Bao, Yong-Sheng Xu

**Affiliations:** ^1^ Orthopedics Center, Inner Mongolia People’s Hospital, Hohhot, China; ^2^ Graduate School, Inner Mongolia Medical University, Hohhot, China

**Keywords:** artificial knee replacement, artificial joint prosthesis, finite element analysis, bionics, biomechanics, kinematics, prosthesis design

## Abstract

Artificial knee arthroplasty, as the most effective method for the treatment of end-stage joint diseases such as knee osteoarthritis and rheumatoid arthritis, is widely used in the field of joint surgery. At present, Finite element analysis (FEA) has been widely used in artificial knee replacement biomechanical research. This review presents the current hotspots for the application of FEA in the field of artificial knee replacement by reviewing the existing research literature and, by comparison, summarizes guidance and recommendations for artificial knee replacement surgery. We believe that lower contact stress can produce less wear and complications when components move against each other, in the process of total knee arthroplasty (TKA), mobile-bearing prostheses reduce the contact surface stress of the tibial-femoral joint compared with fixed-bearing prostheses, thus reducing the wear of the polyethylene insert. Compared with mechanical alignment, kinematic alignment reduces the maximum stress and maximum strain of the femoral component and polyethylene insert in TKA, and the lower stress reduces the wear of the joint contact surface and prolongs the life of the prosthesis. In the unicompartmental knee arthroplasty (UKA), the femoral and tibial components of mobile-bearing prostheses have better conformity, which can reduce the wear of the components, while local stress concentration caused by excessive overconformity of fixed-bearing prostheses should be avoided in UKA to prevent accelerated wear of the components, the mobile-bearing prosthesis maintained in the coronal position from 4° varus to 4° valgus and the fixed-bearing prosthesis implanted in the neutral position (0°) are recommended. In revision total knee arthroplasty (RTKA), the stem implant design should maintain the best balance between preserving bone and reducing stress around the prosthesis after implantation. Compared with cemented stems, cementless press-fit femoral stems show higher fretting, for tibial plateau bone defects, porous metal blocks are more effective in stress dispersion. Finally, compared with traditional mechanical research methods, FEA methods can yield relatively accurate simulations, which could compensate for the deficiencies of traditional mechanics in knee joint research. Thus, FEA has great potential for applications in the field of medicine.

## Introduction

FEA is a computer simulation of the physical forces in real working conditions through models and study parameters. In Rybicki et al. (1972) and Brekelmans et al. (1972) applied FEA in orthopaedics for the first time, which greatly promoted the application of FEA technology in orthopaedics. In artificial knee arthroplasty, also known as knee joint surface replacement, artificial biomaterials are used to replace diseased cartilage and bone of the knee joint after worn and damaged components of the articular surface are removed (Price et al., 2018). This procedure is the most effective means for the treatment of end-stage knee osteoarthritis (Ferket et al., 2017; Price et al., 2018). However, biomechanical problems such as prosthesis loosening, periprosthetic fracture and prosthesis wear after knee arthroplasty remain to be solved (Wang et al., 2021). At present, FEA models can be observed at any angle, and operations on the model, such as determining the osteotomy thickness, analysing the stress of different prosthetic materials (Castellarin et al., 2023), and measuring the alignment between prostheses, can be simulated. FEA methods can overcome the shortcomings of traditional mechanical research methods, such as the long cycles, non-repeatable operations and high costs. This paper summarizes the application of FEA in TKA, UKA and RTKA in the context of the operation scheme, prosthesis design and material selection to provide a reference for biomechanical research on knee arthroplasty.

## Application of FEA in TKA

The option of TKA provides an effective means of functional reconstruction in patients with severe physical knee dysfunction (Carr et al., 2012). Although the process of TKA is becoming increasingly mature, surgeons also remain troubled by postoperative prosthesis loosening, periprosthetic fracture, and prosthesis wear due to a poor knee mechanical environment. FEA can simulate the occurrence of these problems in a computer. Much research has been conducted, focus mainly on prosthesis material and component design and component alignment, among other factors (Innocenti et al., 2016; Park et al., 2021).

### Application of FEA in prosthesis material and design

According to the mode of connection between the polyethylene insert and metal tibial component, TKA prostheses can be divided into fixed- and mobile-bearing prostheses based on whether the polyethylene insert and metal tibial component are locked. In fixed-bearing prostheses, the polyethylene insert is fixed to the tibial component through a locking mechanism; in mobile-bearing prostheses, a movable joint is formed with the femoral component, and a certain degree of movement between the polyethylene insert and the tibial component is allowed, comparing the two designs, it was found that the former insert allows for longitudinal rotation of the tibial or allows for anterior-posterior displacement between the insert and the tibial. Due to the rotation and displacement effect between the mobile insert and the tibial component, the tibial component can better fit the femoral component without sacrificing the natural rotation and displacement between the tibial and the femur, in fixed-bearing prostheses, the polyethylene insert locks on the tibial component, limiting the relative motion between the components (Ranawat et al., 2004). Depending on the degree of restriction on the knee joint, TKA prostheses can be divided into constrained condylar knee (CCK) prosthesis and the rotating hinge knee (RHK). CCK is designed to restrict knee motion through the postcam and femoral box and is considered a semirestricted prosthesis (Andreani et al., 2020; Athwal et al., 2021). RHK connects the femoral prosthesis to the tibial prosthesis via a rotating shaft, thereby achieving maximum restraint (Clement et al., 2023). The contact pressure and contact area of the tibiofemoral joint after TKA are related to the prosthesis design, with the contact stress being inversely proportional to the contact area. Greenwald and Heim (2005) compared the tibial-thigh contact area of fixed- and mobile-bearing prostheses by three-dimensional finite element gait analysis. The results showed that the contact area of the mobile-bearing prosthesis polyethylene insert was 400–800 mm^2^, while that of the fixed-bearing prosthesis polyethylene insert was 200–250 mm^2^. The findings confirmed that the contact area of the tibiofemoral joint of the mobile-bearing prosthesis was higher than that of the fixed-bearing prosthesis; thus, the contact stress of the mobile-bearing prosthesis was lower than that of the fixed-bearing prosthesis because the contact pressure was inversely proportional to the contact area Castellarin et al. (2023) (Table 1). Stukenborg-Colsman et al. (2002) performed an FEA study on cadavers and reached the same conclusion: compared with fixed-bearing prostheses, mobile-bearing prostheses maximized the contact area of the tibiofemoral joint and reduced the peak contact pressure, so that the mobile-bearing prosthesis polyethylene insert provides more movement for the prosthesis and minimizes polyethylene wear compared to the fixed-bearing prosthesis model (Heesterbeek et al., 2018; Andreani et al., 2020), resulting in improved implant survival and performance. For CCK and RHK prostheses, Samiezadeh et al. (2019) performed FEA on both and found that the CCK prosthesis should produce 16.9 MPa interprosthetic stress at the bone-prosthesis interface of 37.6 MPa, while the RHK bone-prosthesis interface stress was 13.7 MPa, a decrease of 18.9%, and the average stress of the CCK polyethylene insert was 9.6 MPa, while the RHK was 2.6 MPa, a decrease of 72.7%.

**TABLE 1:** Application of FEA in TKA.

Refence	Experimental design	Conclusion
Greenwald and Heim (2005)	Fixed-bearing prosthesis vs. mobile-bearing prosthesis	The tibiofemoral joint contact area of mobile-bearing prostheses is higher than that of fixed-bearing prostheses. The contact stress of mobile-bearing prostheses is lower than that of fixed-bearing prostheses.
Stukenborg-Colsman et al. (2002)	Fixed-bearing prosthesis vs. mobile-bearing prosthesis	Compared with fixed-bearing prostheses, mobile-bearing prostheses maximize the tibiofemoral joint contact area and reduce the peak contact pressure. The greater the tibiofemoral joint contact stress, the more serious the wear of the polyethylene insert.
Brihault et al. (2016)	All-polyethylene tibial component vs. metal-backed tibial component	The stress distribution of all-polyethylene tibial components under the plateau is obviously higher than that of metal-backed tibial components.
Tokunaga et al. (2016)	All-polyethylene tibial component vs. metal-backed tibial component	The stress of metal tibial components is lower than that of all-polyethylene tibial components, and the stress distribution is more balanced.
Castellarin et al. (2019)	Asymmetric insert vs. symmetric insert	During standing and squatting movements, lower tibial contact stress was observed in those with asymmetric spacers than those with symmetric spacers.
Klasan et al. (2022)	Kinematic alignment vs. mechanical alignment	Lower contact pressure on the polyethylene insert was observed with kinematic alignment than mechanical alignment.
Song et al. (2021)	Kinematic alignment vs. mechanical alignment	Lower contact pressure on the polyethylene insert was observed with kinematic alignment group than mechanical alignment.

In summary, the low degree of matching achieved with a fixed-bearing prosthesis can reduce the stress at the interface between the knee joint bone and prosthesis and reduce loosening of the prosthesis, but at the cost of relatively high contact pressure with the knee joint and increased wear of the polyethylene insert. The contradiction between free rotation and low joint contact pressure is a problem that cannot be solved by fixed-bearing prostheses. The high degree of matching achieved with mobile-bearing prostheses can reduce the stress at the interface between the knee joint bone and the prosthesis and reduce the contact pressure, thus reducing the wear of the polyethylene insert; additionally, mobile-bearing prostheses can rotate freely, which can reduce loosening of the prosthesis and compensate for this disadvantage of fixed-plateau prostheses. At present, most FEA studies have shown that mobile-bearing prostheses can improve the degree of joint matching, reduce the contact surface stress of the tibiofemoral joint, and thus reduce polyethylene wear. However, the postoperative efficacy and prosthesis survival rate after the clinical application of the two types of prostheses have not been compared in this context, and further long-term follow-up studies are required (Hantouly et al., 2022). For CCK and RHK prostheses, the latter is better in terms of stress performance, and the 2-year clinical follow-up also confirms that the use of RHK provides good results compared to CCK (Hermans et al., 2019).

**FIGURE 1:**
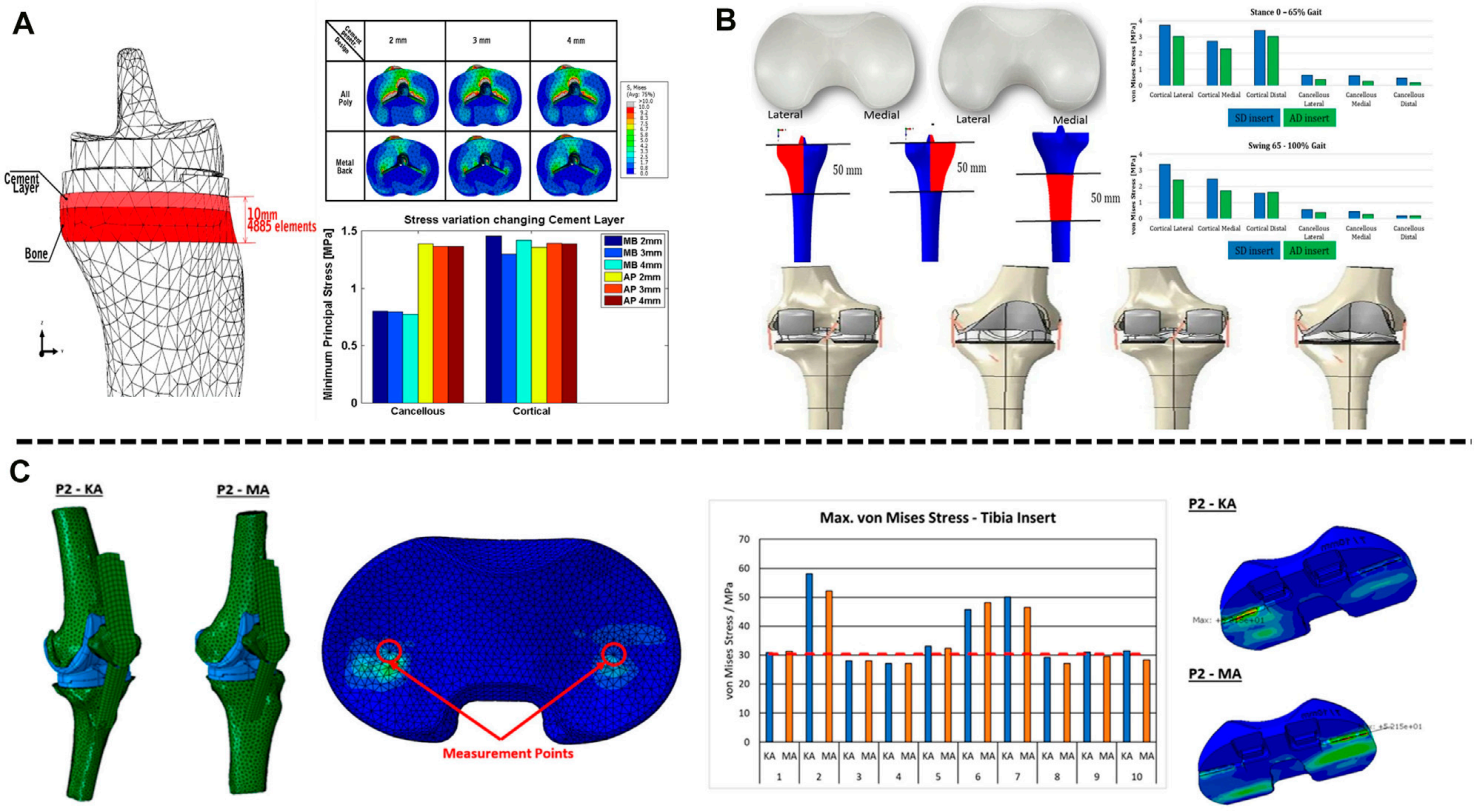
Application of FEA in TKA. **(A)** Contact stress behaviour of all-polyethylene and metal backed-tibial components at the proximal tibia. **(B)** Mechanical difference between symmetric and asymmetric inserts in the proximal tibial cortex. **(C)** Effects of mechanical alignment and kinematic alignment on the surface contact stress of inserts in TKA. **(A)** Reprinted with permission from ref. Brihault et al. (2016) (Copyright 2023; Springer Nature Switzerland AG.). **(B)** Reprinted with permission from ref. Castellarin et al. (2019) (Copyright 2023; Springer Nature Switzerland AG). **(C)** Reprinted with permission from ref. Klasan et al. (2022) (Copyright 2022; MDPI, Basel, Switzerland).

Artificial joint prostheses of different materials show different biomechanical characteristics in artificial joint replacement. Current tibial components consist of two main material options, all-polyethylene tibial components and metal-backed tibial components, which differ in material and component method, resulting in differences in prosthesis-bone and component-component stresses. It was found that while a significant increase in measured strain was observed for both the all-polyethylene and metal-backed tibial components in the simulated load distribution, the all-polyethylene tibial component showed a more pronounced stress rise at the proximal tibia (Small et al., 2010). Brihault et al. (2016) compared the stress distribution and fretting of tibial plateau prostheses made of different materials when the knee joint flexed 120°. The research results showed that the stress distribution of the all-polyethylene tibial component under the plateau was significantly higher than that of the metal-backed tibial component, with fretting five times higher than that of the metal-backed tibial component (Figure 1A). Tokunaga et al. (2016) established a standing finite element model to compare the stress distribution of the metal-backed tibial component and all-polyethylene tibial component. The results showed a significant increase in proximal tibial cortical stresses in the standing alignment after implantation of the all-polyethylene tibial component and a posterior shift in tibial loading with increased resection depth. For clinical outcomes, metal-backed tibial components and all-polyethylene tibial components did not show any significant differences in most of the outcome scores included, but statistically significant differences were found in complication and revision rates. A meta-analysis showed a revision rate of 1.85% in the metal-backed tibial component group compared with 2.02% in the all-polyethylene tibial component group (Longo et al., 2017). Surgeons tend to prefer metal-backed prostheses when selecting the appropriate tibial component for their patients. With advances in material science, all-polyethylene tibial components will have better mechanical properties and value for future applications given their cost and modular design advantages (Gioe and Maheshwari, 2010; AbuMoussa et al., 2019). The polyethylene insert between the femoral and tibial plateau plays a buffering role in knee joint movement, and polyethylene inserts of different design types have different biomechanical effects. The available polyethylene inserts included symmetrical and asymmetrical designs, and to compare the postoperative mechanical results of both, Castellarin et al. (2019) retrospectively analysed 303 patients treated with TKA by the FEA method. Under the condition of the same tibial and femoral components, symmetric and asymmetric polyethylene inserts were used in 151 and 152 patients, respectively. During standing and squatting, the contact stress in the tibial component was lower in those with asymmetric inserts (Figure 1B), at the 2-year follow-up, the asymmetric polyethylene insert group was able to perform certain routine movements better and without any pain, while patients in the symmetrically designed polyethylene group reported pain, validating the FEA results (Castellarin et al., 2021).

In addition to the material and design of the prosthesis, the type matching of the tibial component also affects the service life of the polyethylene insert after TKA. Completo et al., 2010 constructed a finite element model and paired a No. 3 femoral component with No. 2.5, 3, and 4 tibial components, and the corresponding polyethylene insert size (10 mm) was used to study the stress at each flexion angle. The results showed that the interface stress of the No. 3 femoral component paired with the No. 3 tibial component was significantly higher than that of the No. 4 tibial component. Such increased stress accelerates the wear of the polyethylene insert, which in turn affects the life of the prosthesis.

### Application of FEA in component alignment

The success of TKA is closely related to the recovery of the lower extremity force line (Srivastava et al., 2012; Panni et al., 2018; Johnston et al., 2019). Poor alignment of the lower limbs and misalignment of the prosthesis can lead to abnormal wear of the polyethylene liner and premature loosening of the components, thus affecting the life of the components. The force line of the lower limb is formed by the connection of the centre of the femoral head to the centre of the tibial at the ankle joint. Normally, the axis passes through the centre of the knee joint, which is called the mechanical axis of the neutral position. In a normal knee joint, the tibia is 3° varus and the femur is 9° valgus relative to the force line of the lower extremity, respectively, with respect to the force line, and the midpoint of the tibial intercondylar ridge may shift inwards or outwards with respect to the lower extremity force line during disease (Yilmaz et al., 2016). Current finite element studies on component alignment have focused on component angulation and alignment theory. For tibial component alignment angles, scholars used a fixed-bearing TKA model in computer simulations to investigate tibial stress distribution and component micromovement when tibial components were placed in translation and rotation and found that tibial stress and ligament tension increased when laterally offset, and tibial stress and component micromovement were greatest when reaching 6 mm and that stress and micromovement were acceptable when controlling errors to within 2 mm acceptable (Mizu-Uchi et al., 2022). Mell et al. (2022) predicted by the model that tibial component rotation at 15° alignment can cause severe wear up to 5 mm^3^/million cycles, almost twice as much as neutral alignment. For femoral components, scholars modelled femoral components in −3°, 0°, 3°, 5° and 7° flexion to study patellar contact stress and ligament tension and found that patellar contact stress and ligament tension decreased and knee flexion range increased with 3° flexion alignment (Koh et al., 2022). At present, there are two alignment methods in TKA: including early mechanical alignment (MA) and later kinematic alignment (KA). The traditional view is that the lower limb force line of patients after TKA should be reconstructed to a position where the deviation from the neutral position force line is less than 3°; however, KA holds that osteotomy and prosthesis placement should be based on the motion axis of the patient’s knee joint in the normal or prelesion state. MA aims to restore the mechanical axis of the leg (Insall et al., 1985), whereas KA aligns the rotational axis of the component with the three kinematic axes of the knee joint by aligning the component to the natural joint line (Howell et al., 2010). The two alignment methods have variability in different correction situations. Thus, the artificial knee joint can simulate the normal biomechanical state of the human knee joint as much as possible after the operation. Kang et al. (2020) studied ligament-preserving prostheses and found that KA had better mobility and even stress distribution than MA. Klasan et al. (2022) established a finite element model for 10 patients with knee osteoarthritis to simulate TKA with mechanical alignment and kinematic alignment. The results showed a larger contact area and lower contact pressure on the polyethylene insert in TKA patients treated with kinematic alignment than in those treated with mechanical alignment (Figure 1C), but there was no significant difference in von Mises stress between the polyethylene insert and the tibia. Therefore, the pressure distribution on the contact surface of the artificial joint was more uniform, and the stress was lower, reducing the wear of the joint contact surface and prolonging the life of the prosthesis. In addition, Song et al. (2023) found that when the knee joint was valgus, the contact stress on the polyethylene insert was higher with KA than with MA. Generally, the best method for alignment in TKA remains controversial. Before the emergence of KA, MA was the “gold standard” in TKA. KA can maximize the biomechanics of the knee joint, thus achieving better surgical results and functional recovery. However, when simulating patients with valgus deformity and severe flexion deformity, the contact stresses on the polyethylene surface of the KA prosthesis increased compared to MA, probably because valgus requires excessive soft tissue release for balancing and soft tissue release is not available in the flexion deformity model (Nakamura et al., 2017; Klasan et al., 2022), and further experiments and studies are needed.

## Application of FEA for alignment in UKA

Compared with TKA, UKA is a new type of minimally invasive surgery. In UKA, only the injured surface is replaced, such as damaged cartilage in the medial or lateral compartment of the knee joint (Price and Svard, 2011; Hansen et al., 2019). This technique does not require removal of the anterior and posterior cruciate ligament and retains the proprioceptive sensation and function of the knee joint (Cameron and Jung, 1988; Vince and Cyran, 2004). This strategy has the advantages of causing less injury and allowing quick recovery (Scott and Santore, 1981; Berend et al., 2005). However, UKA is a technically demanding surgical method, and attention must be paid to the size of the components, the osteotomy and postoperative alignment because overcorrection or overloosening may lead to adverse results, these include primarily bearing dislocations (in mobile-bearing designs), aseptic mechanical loosening, polyethylene wear (in fixed-bearing designs), progression of osteoarthritis in unreplaced compartments, periprosthetic fractures and unexplained pain (Shee et al., 1990; Kim et al., 2016). Resulting in a typical 10-year survival rate of 80% to 85% for the prosthesis (Crawford et al., 2020), and ways to reduce these adverse events to improve prosthesis longevity need to be continuously explored.

**FIGURE 2:**
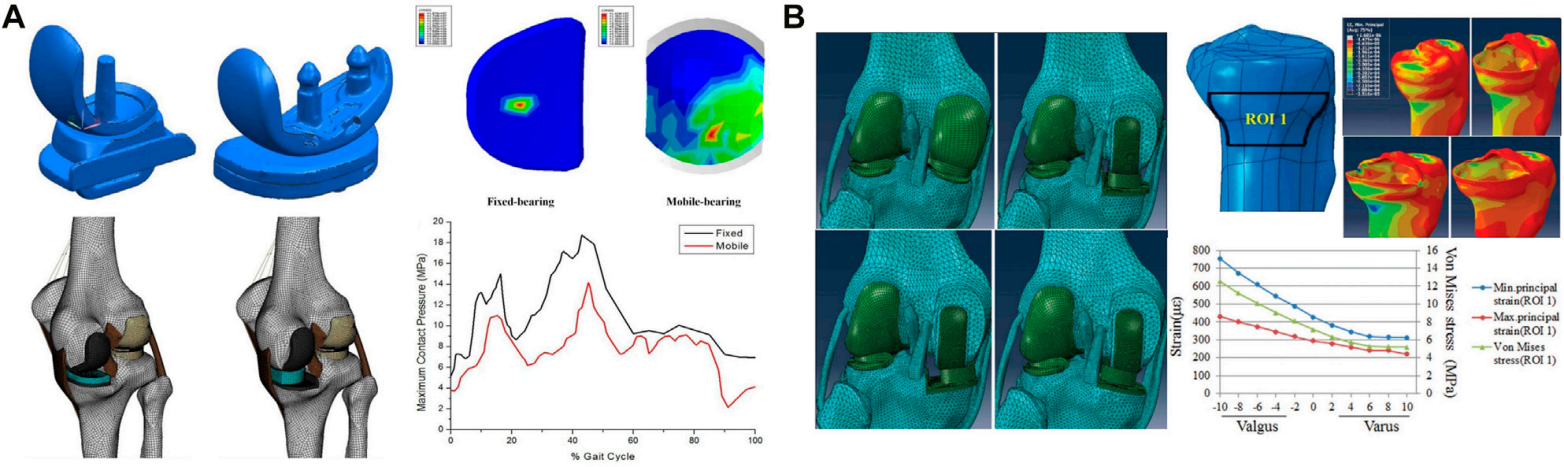
Application of FEA in UKA. **(A)** Comparison of the effect of fixed- and mobile-bearing UKA prostheses on the surface stress of the insert. **(B)** Stress analysis of the proximal tibial cortex under the condition of varus and valgus UKA tibial components. **(A)** Reprinted with permission from ref. Kwon et al. (2014) (Copyright 1999-2023; John Wiley & Sons, Inc.). **(B)** Reprinted with permission from ref. Zhu et al. (2015) (Copyright 2023; The Chinese Medical Association).

Similar to the TKA prosthesis design, the UKA prosthesis has both fixed- and mobile-bearing prostheses, with the mobile-bearing prosthesis allowing the femoral condyle to roll on the polyethylene surface and allowing the polyethylene insert to slide freely on the surface of the tibial component; the fixed-bearing prosthesis locks the polyethylene insert to the tibial component, more closely resembling the movement of the femoral condyle on the meniscal surface (Weale et al., 2000; Hernigou and Deschamps, 2004). For medial compartment osteoarthritis, both prostheses have good clinical results (Neufeld et al., 2018). Mobile-bearing prostheses use the spherical articular surface to limit the range of motion of the femur relative to the articular surface while using a curved surface with the same curvature to maximize the contact surface (Figure 2A) and reduce the pressure on the lateral meniscus by 1/3 (Kwon et al., 2014). In contrast, the design concept of fixed-bearing prostheses is the opposite. Fixed-bearing prostheses more closely imitate the motion mode of the normal knee joint by setting the active area between the femoral component and the polyethylene insert. Kwon et al. (2014) used a finite element model to simulate the complete gait and compare the contact pressure and stress of fixed-bearing and mobile-bearing prostheses. While the results showed that the contact pressure on the polyethylene insert was lower for mobile-bearing prostheses than fixed-bearing prostheses, UKA with fixed-bearing prostheses will increase the overall risk of progressive knee osteoarthritis due to the high pressure in the contralateral chamber. Danese et al. (2019) and Sano et al. (2020) relied on FEA to investigate the stress distribution in the material and shape of the prosthesis. It was found that fixed-bearing prostheses may lead to excessive wear of polyethylene inserts due to local stress concentrations and that mobile-bearing prostheses are closer to the normal knee joint (Koh et al., 2019; Koh et al., 2020), a finding confirmed by studies by Pena et al. (2006) and Li et al. (2006). Zhu et al. (2015) studied the coronal arrangement of the tibial component on mobile-bearing prostheses. The von Mises stress and compressive strain of the proximal part of the medial tibial cortex increased significantly when the tibial component was more than 4° valgus, and the compressive strain at the keel notch of the tibial prosthesis was higher than the maximum threshold when the tibial component was more than 4° varus (Figure 2B). Therefore, Dai et al. (2018) verified the effect of bone stress, ligament tension and polyethylene liner stress distribution in the tibial component from neutral to 6° varus by using a finite element model of mobile-bearing prosthesis and found that neutral alignment to 3° varus exhibited lower stresses, which supported the above findings, the recommended angle of the tibial component of mobile-bearing prostheses on the coronal plane is between 4° varus and 4° valgus. Innocenti et al. (2016) studied the coronal plane of fixed-bearing prostheses and established a model in which the femoral and tibial components had different varus and valgus angles while the posterior inclination of the tibial component was 6°. The results showed that during the gait cycle, the stress caused by tibial component varus and valgus on the polyethylene insert was greater than that in the neutral position, Sekiguchi et al. (2019) simulated knee kinematics and cruciate ligament tension in weight-bearing knee flexion and gait motion and found that the preferred tibial component alignment was neutral in the coronal plane and that varus or valgus alignment resulted in the onset of instability. Kang et al. (2018b) observed that as the fixed-bearing UKA femoral component was progressively valgus from a neutral alignment, the contact pressure on the polyethylene insert increased, and the contact stress on the lateral compartment also increased. Therefore, it is recommended that the femoral component be placed in neutral alignment. Ma et al. (2022) simulated the stress changes on the polyethylene insert and the cartilage surface of the lateral compartment for femoral components with 3°, 6° and 9° of valgus and found that the stresses on the polyethylene surface increased significantly when the femoral prosthesis was valgus, the stresses on the lateral cartilage surface and the medial collateral ligament increased significantly at >6° (Kang et al., 2018a), and the stresses on the lateral intertrochanteric cartilage surface decreased during the transition from valgus to neutral alignment, thus confirming the above findings. Park et al. (2019) modelled a fixed-bearing UKA femoral component in the range from 10° of flexion to 10° of extension and found that the lateral intertrochanteric contact stresses increased in flexion, suggesting that the femoral component be placed in the sagittal neutral alignment. Thus, in terms of the coronal arrangement of the tibial component of fixed-bearing prostheses, the neutral position (0°) is recommended.

In summary, FEA results show that good shape matching can be achieved with the femoral and tibial components of mobile-bearing prostheses, which can reduce the wear of the components, while fixed-bearing prostheses should be avoid in UKA to prevent the local stress concentration and subsequently accelerated insert wear caused by excessive fitting. Additionally, the FEA shows that for optimal clinical and biomechanical results, fixed-bearing prostheses should be placed with the tibial component in a neutral (0°) alignment and the femoral component in a neutral alignment while maintaining a reduced sagittal plane flexion angle. For mobile-bearing prostheses, 4° varus to 4° valgus alignment is recommended by scholars.

Materials included metal-backed and all-polyethylene tibial components. Walker et al. (2011) found a stress concentration at the proximal tibia due to the all-polyethylene component by comparing all-polyethylene and metal-backed tibial components. In contrast, the metal-backed tibial component showed a 6-fold reduction in stress. Sano et al. (2020) compared the stresses in the proximal tibia with the metal-backed UKA prosthesis by FEA, and with consistent bone density and alignment, the metal-backed implant had a stress distribution that was more uniform than that of polyethylene, reducing the stress concentration in the proximal tibial cortex. Koh et al. (2019) compared the polyethylene surface contact stresses of the two prostheses and found that the metal-backed component reduced the contact stresses and had lower wear (Table 2).

**TABLE 2:** Application of FEA in UKA.

Refence	Experimental design	Conclusion
Zhu et al. (2015)	Mobile-bearing prosthesis	The recommended angle of coronal alignment of the tibial component is between 4° varus and 4° valgus.
Ma et al. (2022)	Fixed-bearing prosthesis	The coronal arrangement of the femoral component should be neutral (0°).
Innocenti et al. (2016)	Fixed-bearing prosthesis	The coronal arrangement of the tibial component should be neutral (0°).
Kwon et al. (2014)	Fixed-bearing prosthesis vs. mobile-bearing prosthesis	The contact pressure of the polyethylene liner is lower on mobile-bearing prostheses than on fixed-bearing prostheses.
Koh et al. (2019)	Fixed-bearing prosthesis vs. mobile-bearing prosthesis	The contact pressure of the polyethylene liner is lower on mobile-bearing prostheses than fixed-bearing prostheses.
Sano et al. (2020)	All-polyethylene tibial component vs. metal-backed tibial component	The stress of metal tibial components is lower than that of all-polyethylene tibial components, and the stress distribution is more balanced.

## Application of FEA in RTKA

RTKA is expected to increase with the increase in primary knee arthroplasty for the active patient population (Lombardi et al., 2019). RTKA is associated with high operative difficulty, great trauma to patients, long recovery periods and complex clinical management in the perioperative period, is a challenging procedure with often unsatisfactory outcomes compared to primary knee arthroplasty (Bae et al., 2013). Due to the rapid increase in the number of patients treated with primary knee arthroplasty annually, RTKA has gradually become the focus of future research in the field of joint surgery (Mason and Fehring, 2006). The causes of surgical failure have been studied by FEA, and the design of procedures and prostheses used in RTKA have been further optimized from a biomechanical point of view. Understanding the effects of materials and prosthesis design from a biomechanical perspective is the current focus of FEA.

### Application of FEA in stem implant design

**FIGURE 3:**
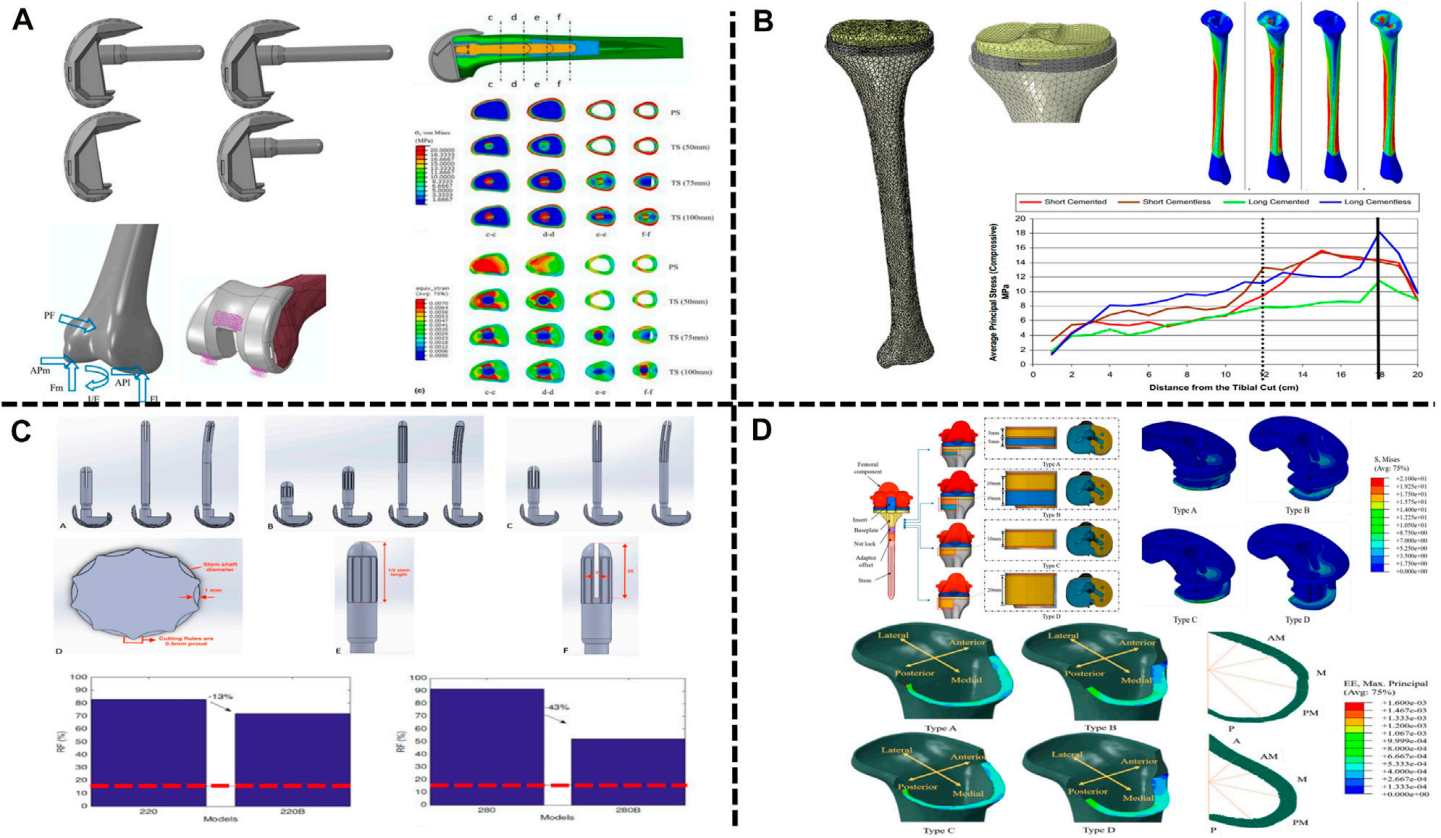
Application of FEA in RTKA. **(A)** Contact stress of different types of femoral implants. **(B)** Cortical bone stress of cemented and cementless press-fit tibial components. **(C)** Comparison between straight and bowed stem implants. **(D)** RTKA bone defect metal augmentation block design. **(A)** Reprinted with permission from ref. Conlisk et al. (2018) (Copyright 2023; Springer Nature Switzerland AG). **(B)** Reprinted with permission from ref. El-Zayat et al. (2016) (Copyright 2023; Springer Nature Switzerland AG). **(C)** Reprinted with permission from ref. Innocenti et al. (2022) (Copyright 2022; MDPI, Basel, Switzerland). **(D)** Reprinted with permission from ref. Kang et al. (2019) (Copyright 2022; MDPI, Basel, Switzerland).

Adequate fixation is the basis of RTKA, and modular implants with stems can reduce stress concentrations and improve the outcome. However, there are no biomechanical-based guidelines to determine the appropriate stem length, according to the fixation mode, prostheses can be divided into cemented and cementless prostheses (cementless press-fit prostheses). The femoral condylar surface of most prostheses is fixed with bone cement, and the stem implant can be fixed with bone cement or compression without cement. Cementless prostheses can be more easily revised, but cemented prostheses should be used in patients with obvious osteoporosis or metaphyseal deformity. Conlisk et al. (2018) analysed cemented prostheses with the finite element method. They constructed four kinds of femoral components with different lengths, namely, a femoral component without a stem implant and with a short-stem implant (50 mm), a medium-stem implant (75 mm) and a long-stem implant (100 mm) (Figure 3A). Bori et al. (2022) considered that a medium-length stem (75 mm) is the best choice between bone preservation and stress reduction, especially in osteoporotic patients, and can help reduce the risk of periprosthetic fractures. Each stem implant was implanted into the femur, and the stress and strain around the femur were analysed during the gait cycle (Wang et al., 2021). The use of components with stem implants could reduce the stress around the prosthesis, and the stress on the femur could be reduced by 11%, 26%, and 29% with the use of the short-, medium- and long-stem implants, especially in osteoporotic patients, and can help reduce the risk of periprosthetic fracturesth (Bori et al., 2022). However, in the combined case of preserving bone and balancing stresses, medium-stem implants (75 mm) are considered to maintain the best balance between preserving bone during surgery and reducing stress around the prosthesis after implantation, especially in osteoporotic patients, and can help reduce the risk of periprosthetic fracturesth (Bori et al., 2022) (Table 3).

**TABLE 3:** Application of FEA in RTKA.

Refence	Experimental design	Conclusion
Conlisk et al. (2018)	Comparison of the effects of short-, medium-, and long-stem implants	The femoral stress was decreased by 11%, 26%, and 29% with short-, medium-, and long-stem implants, respectively. A medium-length (75 mm) femoral stem implant can keep the best balance between retaining bone and reducing stress around the prosthesis after implantation.
El-Zayat et al. (2016)	Press-fit stem implant vs. cemented stem implant	Compared with cemented prostheses, during the gait cycle and during squatting, press-fit stem implants resulted in a larger stress distribution and more easily led to fretting.
Innocenti et al. (2022)	Straight stem implant vs. bowed stem implant	The overall stress distribution of bowed stem implants is lower than that of straight stem implants.
Innocenti et al. (2018)	Bone cement vs. porous metal vs. solid metal	The stress produced by porous metal and bone cement is significantly lower than that produced by solid metal (e.g., Ti and CoCr). In addition, the characteristic of porous metal allowing bone ingrowth further reduces the occurrence of loosening.
Kang et al. (2019)	Single-metal block augmentation vs. double metal block augmentation	Bimetal augmentation blocks can obviously reduce the stress distribution compared with single-metal augmentation blocks.

El-Zayat et al. (2016) compared the stress distribution on the femur between cemented and cementless stem implants by FEA. Compared with the cemented component, found that the presence of cement reduced peri-stem bone stress during the gait cycle, while the cementless stem showed higher micromotion, which may explain pain at the tip of the intramedullary stem implant reported after RTKA (Figure 3B). Innocenti et al. (2022) further analysed the influence of the shape of the stem implant on the stress distribution with the use of different fixation methods on the basis of the study by El-Zayat et al. (2016). It was found that due to the existence of the anterior femoral arch, the overall stress distribution of the bowed stem implant was lower than that of the straight stem implant; intramedullary stems with a slotted tip also resulted in lower stress than solid stems in the medullary cavity (Figure 3C). These findings are consistent with those reported by Barrack et al. (2004). In RTKA, the presence of bone cement reduces the stress along each area of the prosthesis. In summary, the length of the stem implant should maintain the best balance between preserving the bone and reducing the stress around the prosthesis after implantation, and compared with cemented stem implants, press-fit stem implants show more fretting. In addition, bowed stem implants more closely mimic the anatomical structure, and the use of stems with a slotted tip can help reduce the stress distribution at the distal end and reduce the occurrence of tip pain after the operation. These findings will help orthopaedic surgeons select the most suitable prosthesis for use in RTKA.

### Application of FEA in augmentation block material selection

Augmentation blocks are one of the options for the reconstruction of nonenclosed bone defects, and the technique is based on the size of the defect, the patient’s age and life expectancy (Qiu et al., 2012), but the mechanical performance varies between materials. Innocenti et al. (2018) and Liu et al. (2021) used a finite element model to analyse the biomechanics of augmentation blocks of different materials (bone cement, porous metal and solid metal). The results showed that augmentation blocks of any material can cause a change in stress, especially in the area near the bone defect, in which the stress produced by porous metal and bone cement is significantly lower than that produced by solid metal (e.g., Ti and CoCr). In addition, porous metal allows bone ingrowth, which further reduces the occurrence of loosening. Kang et al. (2019) further found that in large-area (10–20 mm) bone defects, compared with a single-metal augmentation block, found that the peak stress of a single-metal augmentation block was on average 1.4 times higher than that of bimetal augmentation blocks. In addition, customized metal augmentation blocks can achieve complete contact with cortical bone, thereby allowing better stress transfer and reducing the risk of bone resorption caused by stress shielding and cement failure (Figure 3D). At present, the main methods for the treatment of bone defects in RTKA include bone cement, bone cement with screw reinforcement, metal augmentation blocks, pressed bone transplantation and structural allografting (Huten et al., 2021), depending on the location and size of the defect (Mancuso et al., 2017). Comparing the cement-screw technique and metal augmentation block, Zheng et al. (2020) determined the role of screws in repair by building a finite element model of tibial bone defects and found that, compared with bone cement alone, the use of cement screws decreased stresses on the cancellous bone and cement boundary by 10%, while vertical screws provided better stability than oblique screws. Zhao et al. (2022) found that vertical screws had better stability than screws parallel to the proximal tibial cortical bone for either one or two screws, supporting the above findings. Ma et al. (2023) found that longer screws may not achieve better stability with consistent defect conditions, but thicker screws reduced stresses in the area of the bone defect and achieved better stability. Liu et al. (2020) modelled 5 mm and 10 mm bone defects and found that for 5 mm defects, both methods provided good stability for the implants. However, for the 10 mm defect, the maximum micromovement of the augmentation block (128 μm) was less than that of the cement-screw technique (155 μm). Because metal augmentation blocks are easy to use and transfer stress well, they are increasingly used in the treatment of bone defects. In addition, basis of porous metal augmentation blocks, scholars suggest that the biomechanical properties of bone can be improved by tailoring the shape to reduce bone resorption (Liu et al., 2021), customized metal augmentation blocks can be used to optimize the treatment of bone defects (Bao et al., 2013) (Table 3).

## Conclusion and outlook

We have compared different surgical approaches, prosthesis types, prosthesis materials and component alignments by summarizing the current applications of finite element techniques in the field of TKA, UKA and RTKA to provide a mechanical theoretical and research reference for clinical purposes. By reviewing the above studies, it was found that the contact surface stress of the tibiofemoral joint is lower with mobile-bearing prostheses than fixed-bearing prostheses in TKA and thus reduces polyethylene insert wear. Compared with mechanical alignment, TKA with kinematic alignment reduces the maximum stress and strain of the femoral component and polyethylene insert and thus reduces the wear of the joint contact surface and prolongs the life of the prosthesis. However, the gap in the practical application of the two is relatively small in current clinical research, and further study is needed. Asymmetric metal-backed tibial components are less stressful between components and to the underside of the tibial plateau than symmetric all polyethylene tibial components. In addition, mismatching of the prosthesis type leads to an increase in stress, accelerates the wear of the polyethylene insert and affects the service life of the prosthesis. In UKA, while the femoral and tibial components of mobile-bearing prostheses are more formable, which can reduce the wear of the prosthesis insert, fixed-bearing prostheses should be avoided to prevent the local stress concentration and subsequently accelerated wear caused by excessive formation. From an FEA perspective, it is recommended that the tibial component of the mobile-bearing prostheses is arranged from 4° varus to 4° valgus on the coronal plane, while that of fixed-bearing prostheses should maintain a neutral position (0°). In RTKA, the length of the stem should maintain the best balance between preserving bone and reducing stress around the prosthesis after implantation, a 95 mm to 100 mm stem implant can help with better fixation, but a medium stem (75 mm) is more appropriate to preserve as much bone as possible. Compared with the cemented stem implants, press-fit stem implants show more fretting. In addition, bowed stem implants are more similar in shape to the anatomical structure, and stems with slotted tips in the medullary cavity are beneficial for reducing the stress distribution at the distal end, thus reducing the occurrence of tip pain after surgery. For periprosthetic bone defect repair, should take into account the location and size of the defect, and using the cement-screw technique is more suitable for smaller defects. If the defect is larger, double porous metal augmentation blocks are more appropriate.

In the future, FEA can be used to carry out relatively accurate calculations and simulations in knee arthroplasty; FEA has been further studied from the perspectives of prosthesis material and design, knee joint alignment and operation scheme, provide an important reference for the accurate diagnosis and treatment of knee diseases, the design of artificial knee prosthesis and the study of knee biomechanics.
